# Assessing cognitive decline in the aging brain: lessons from rodent and human studies

**DOI:** 10.1038/s41514-023-00120-6

**Published:** 2023-10-19

**Authors:** D. V. C. Brito, F. Esteves, A. T. Rajado, N. Silva, ALFA score Consortium, R. Andrade, J. Apolónio, S. Calado, L. Faleiro, C. Matos, N. Marques, A. Marreiros, H. Nzwalo, S. Pais, I. Palmeirim, V. Roberto, S. Simão, N. Joaquim, R. Miranda, A. Pêgas, D. M. Raposo, A. Sardo, I. Araújo, J. Bragança, P. Castelo-Branco, C. Nóbrega

**Affiliations:** 1 Algarve Biomedical Center-Research Institute (ABC-RI), Campus Gambelas, Bld.2, Faro, Portugal; 2 Algarve Biomedical Center- (ABC), Campus Gambelas, Bld.2, Faro, Portugal; 3 Faculty of Medicine and Biomedical Sciences (FMCB), University of Algarve, Gambelas Campus, Bld.2, Faro, Portugal; 4 Champalimaud Research Program, Champalimaud Centre for the Unknown, Lisbon, Portugal; 5 Faculty of Science and Technology (FCT), University of Algarve, Gambelas Campus, Bld.8, Faro, Portugal; 6 USF Ossónoba, Faro, Portugal; 7 USF Balsa, Tavira, Portugal; 8 USF Mirante, Olhão, Portugal

**Keywords:** Translational research, Cognitive ageing

## Abstract

As life expectancy continues to increase worldwide, age-related dysfunction will largely impact our societies in the future. Aging is well established to promote the deterioration of cognitive function and is the primary risk factor for the development of prevalent neurological disorders. Even in the absence of dementia, age-related cognitive decline impacts specific types of memories and brain structures in humans and animal models. Despite this, preclinical and clinical studies that investigate age-related changes in brain physiology often use largely different methods, which hinders the translational potential of findings. This review seeks to integrate what is known about age-related changes in the brain with analogue cognitive tests used in humans and rodent studies, ranging from “pen and paper” tests to virtual-reality-based paradigms. Finally, we draw parallels between the behavior paradigms used in research compared to the enrollment into clinical trials that aim to study age-related cognitive decline.

## Introduction

One of the prime accomplishments of modern societies is the steady increase in longevity. In one century alone, we have evolved from almost no countries with life expectancies over 50 years, to currently having over 40 countries with life expectancies that exceed 80 years of age^1^. The steady increase in life expectancy was projected to globally slowdown in the latter half of this century, compared to the increase observed from 1990 to 2017^2^. This deceleration is predicted to be more pronounced in countries that already show high life expectancies, than in developing countries. Therefore, estimations towards the end of the century point to an overall global convergence of life expectancies^2^.

The increase of chronological age is generally accompanied by several factors that impair the health quality during the final years of life. Older populations show a higher risk and prevalence of age-associated disorders such as cancer, arthritis, heart, and neurodegenerative disorders^3^. Accordingly, it is well established that cognitive function is affected during aging, as about 40% of individuals aged 65 years or above suffer from some form of memory loss^4–7^. While it is widely acknowledged that aging is the primary risk factor for the development of progressive neurodegenerative disorders like Alzheimer’s disease (AD), it is important to note that the neurobiological changes that occur during aging which result in cognitive deficits are vastly distinct from those observed in AD. For instance, while both aging and AD are associated with brain volume loss, research conducted on humans and animals suggests that the atrophy observed during normal aging primarily results from synaptic loss, rather than cell loss. In contrast, AD is characterized by significant neuronal and synaptic loss^8^. Several studies have shown that mild cognitive impairment (MCI) affects approximately 16% of individuals over the age of 70, while around 14% of people in the same age group experience dementia^9–14^. Furthermore, it is estimated that about 15–20% of patients with MCI may eventually develop dementia^15,16^. Importantly, aged individuals encounter difficulties in performing daily activities and show deterioration as they age, in a gender-specific pattern, even in the absence of disease^17,18^. Notably, the severity of cognitive impairment is strongly linked to hospital admissions^18^. Elderly patients without MCI, but with cognitive impairment, are more prone to frequent hospitalizations compared to those with intact cognitive function^18^. These individuals also face an increased risk of adverse outcomes during their hospital stay^19,20^.

In conclusion, understanding the prevalence and characterizing cognitive impairment spared of disease in the elderly population is crucial to identify and address the healthcare needs of individuals experiencing cognitive decline, as well as for implementing appropriate strategies to mitigate the risk of adverse outcomes during hospitalization^15^.

However, human aging studies are challenging to design and to complete. For example, selection bias in recruiting participants that already display advanced age-related comorbidities can easily result in reduced enrolment in the study. The opposite can also occur, as healthy, active subjects might decline participation in studies due to lack of time^21^. Limited social and/or financial support might also hinder the enrollment to studies and introduce recruitment bias. Lastly, the intrinsic design of aging longitudinal studies may take several decades to complete. The prolonged duration of these studies can lead to many participants opting out before the study concludes, for numerous reasons. These include the inability to participate due to new arising incapacities, geographical relocation or changes in life-style that make them inadequate for the inclusion criteria of the study. These limitations underlie the need for complementary research options to study age-related cognitive decline and associated brain alterations. Animal models show several advantages as they largely decrease variability and allow a more detailed and cost-effective evaluation of the underlying changes associated with age-related cognitive decline. These preclinical studies are also useful to trim down candidate changes and mechanisms to be later evaluated in human studies.

In the past decades, it has become clear that mechanisms leading to cognitive dysfunction associated with aging are largely conserved in a wide range of animal models, such as primates and rodents^22,23^. Therefore, animal models of cognitive aging are highly relevant to complement human aging studies.

**Fig. 1:**
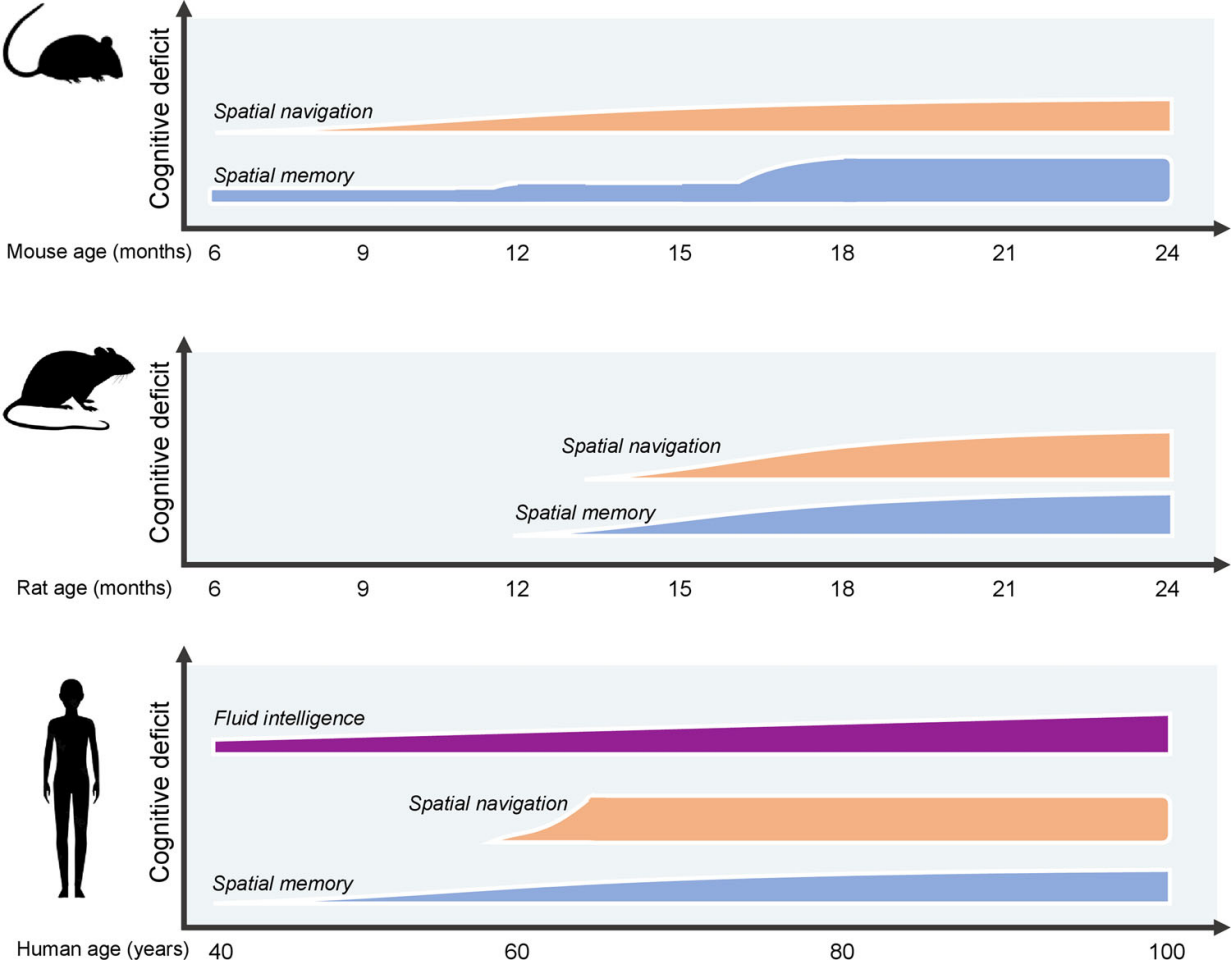
Illustration on the onset and progression of cognitive deficits in humans and rodent animal models. Ages depicted are based on the progression of age-related cognitive decline in mouse^28,122,136–138^, rats^46,114,139,140^ and human^31,117,141,142^ studies.

Animal models of aging, such as rodents, canines, and nonhuman primates, exhibit a decline in cognitive function similar to that observed in humans as they age (Fig. 1). This decline is particularly evident in regions such as the hippocampus, where age-associated memory impairment is observed^24–26^. Interestingly, this decline in memory function in animals is not accompanied by significant neuronal loss, much like in humans. Instead, it appears to be primarily linked to synaptic changes, including the loss of synapses and alterations in synaptic efficacy^27^. An example of this can be seen in the hippocampal long-term potentiation (LTP), a process that mirrors synaptic learning, which becomes more challenging to induce and decays faster in aged animals^28,29^. These changes in animal models during normal aging reflect the translatability of impairments in cognitive performance to observations seen in humans.

The use of an array of animal models is important to investigate the complexity of brain alterations during aging. In particular, rodents share significant genetic and physiological similarities with humans and have a relatively short lifespan compared to larger model organisms. This enables the study of age-related changes and cognitive decline over a shorter time span. Mice and rats also exhibit cognitive abilities and behaviors that are, to some extent, comparable to humans, making them suitable for assessing learning and memory. Moreover, rodents are highly amenable to genetic manipulations, allowing researchers to explore the effects of specific gene modifications or interventions on cognitive aging. Finally, rodent animal models do not spontaneously develop AD-like histopathological hallmarks, which potentially allows the study of age-related cognitive decline without interference from AD-like phenotype^30^.

The use of robust animal models to study cognitive aging remains crucial for understanding the implications of research findings. While human research is undoubtedly the most directly applicable, it faces ethical limitations in terms of manipulation and comprehending the temporal progression of events that could contribute to cognitive impairments during aging. Consequently, translational research aimed at enhancing human health during advanced stages of life relies heavily on the utilization of animal models, specifically rodents and nonhuman primates, to model age-related cognitive decline.

In this review, we will outline the current understanding of cognitive dysfunction in human and rodent models as an effort to facilitate knowledge interchange. This review will focus on specific modifications that occur at a cognitive level, without disease, and behavior analysis conducted in preclinical and clinical settings. We will not focus on molecular and synaptic plasticity modifications that have been described to also occur during the aging process. First, we will discuss the main memory types that are known to be affected during aging, as well as the brain regions that show altered function. Next, we will debate on the methods commonly used to test cognitive performance in humans and animal models, and how they can potentially be compared to better understand the aged brain. Lastly, we will discuss the main methodologies used in age-related clinical trials.

## Cognitive dysfunction throughout aging

Modifications in cognitive performance during aging are well documented in the scientific literature^31,32^. Cognitive abilities are differentially impacted by age, as some are resistant to modifications, while others tend to decrease or even further develop. For example, vocabulary function is particularly unaffected by aging and tends to improves over time^33^. On the other hand, conceptual reasoning, processing speed and memory decline progressively as age advances^34–36^. Cognitive changes are inherent to the aging process, although individuals experience different rates of cognitive decline. However, the acceleration and consequent deterioration of cognitive abilities is linked to the inability to continue daily routines and the development of age-related disorders, such as Alzheimer’s disease and vascular dementia, the two most common forms of dementia that affect the aged population^37^. Moreover, individual levels of cognitive decline are present not only in humans, but also in animal models. This heterogeneity results in good and poor aged performers, which stresses the importance of sensitive cognitive tasks that can accurately distinguish both populations.

### Main types of memory affected throughout aging

**Fig. 2:**
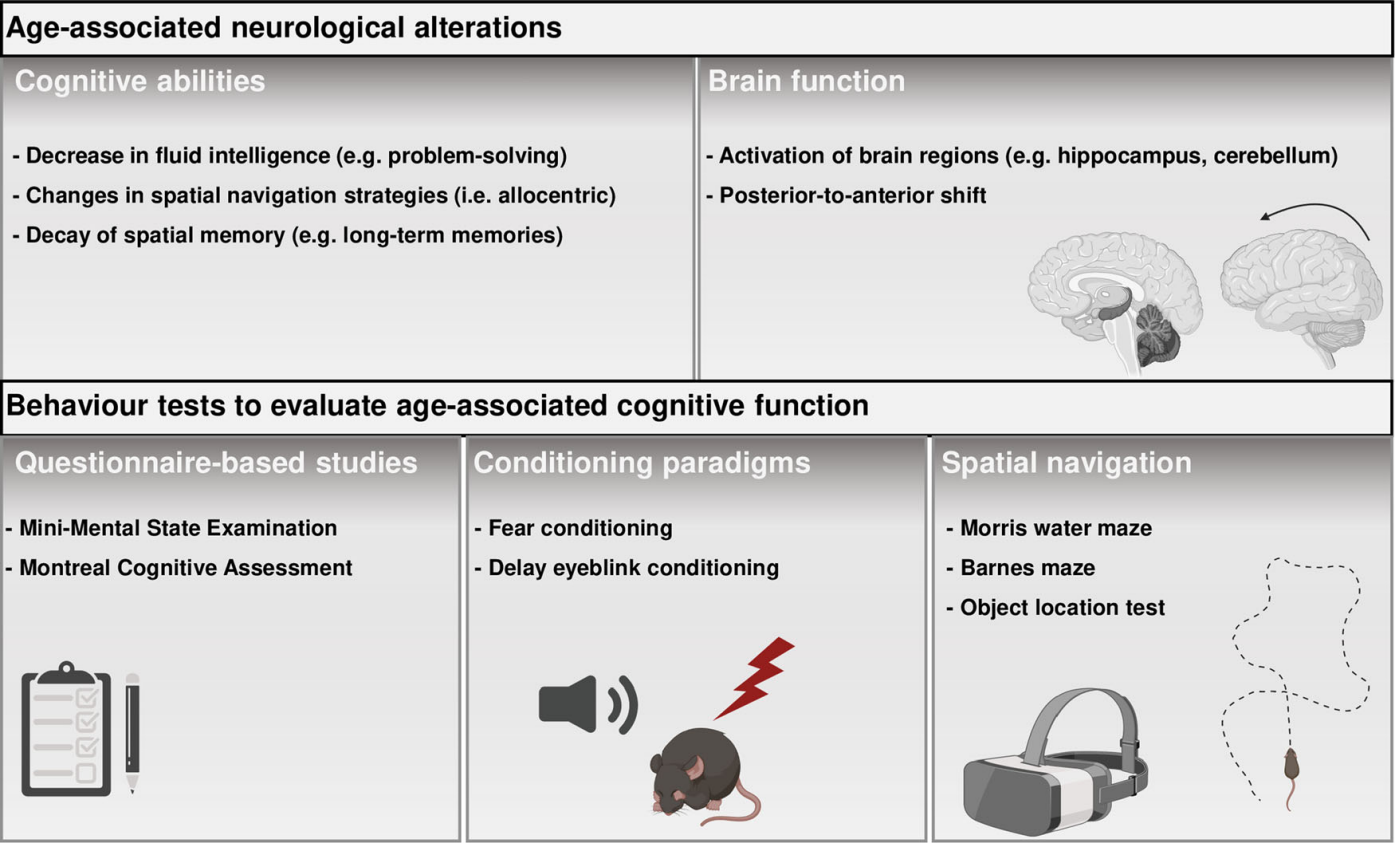
Overview of main neurological alterations during aging and available tests. Major age-associated modifications in cognitive abilities and brain functioning are depicted in the top part of the table. Bottom part highlights “pen and paper” questionaries, routinely performed in the clinic, associative learning and spatial navigation-based tests that can be used to access age-related cognitive decline.

Early psychological research in the 1960s proposed a classification between different types of intelligence, in an attempt to develop scales to evaluate cognitive function^38^. This effort resulted into two types of classification which are still largely used: crystalized intelligence and fluid intelligence^39^. Crystalized intelligence comprises skills that are learned over time. Noteworthy examples include acquisition of vocabulary and general knowledge, that become stable or even increase during aging^31^. Older adults outperform younger adults in tasks that directedly rely on this type of intelligence. Moreover, procedural memories (i.e., how to play an instrument) are largely spared with age. On the contrary, fluid intelligence encompasses abilities of problem-solving and reasoning applied to the processing of novel information, which is less dependent on familiar experiences. Abilities such as processing speed, memory formation and retention peak during early adult age and tend to decrease during aging^40^ (Fig. 2). Interestingly, the formation of new memories declines with age, while autobiographical memories that were acquired in the past tend to be stable, although accuracy for details decline. Divided attention, the ability to learn tasks while simultaneously performing other tasks, a skill that largely affects productivity, is also decreased in the elderly^41,42^.

Although some of these types of memory dysfunction are challenging to evaluate in animal models, such as crystalized intelligence it has become clear that age-related memory impairments are largely conserved across species. Here, we focus mostly on rodent studies, as they are the most commonly used model in neuroscience research. Aged rodents display robust deficits in several types of spatial memories (Fig. 1). Importantly different strains of mice and rats display unique trajectories of age-related cognitive decline onset and progression, which are also gender-specific^43–46^. The formation and consolidation of spatial memories are crucial for navigation abilities and recollection of spatial cues for daily activities^47,48^. Older human adults display impairments on allocentric navigation, which relies on the creation of spatial maps based on the position of each component (e.g., objects) in the environment relative to each other (Fig. 2). As a possible way to cope with these differences, older adults tend to use egocentric strategies for spatial navigation, which rely on creating a spatial representation based on the position of one’s self in relation to the environment. This shift in spatial navigation strategies is likely due to different brain regions that are differently impacted during aging^49^. It is currently thought that these strategies rely on different brain circuitry, particularly allocentric navigation is largely hippocampus dependent, while egocentric navigation is mostly striatal-dependent^50–52^. Alike aged humans, rodents also show this age-related shift in allocentric to egocentric strategies, which provide a model for understanding the cellular and molecular mechanisms underlying this change^53^.

Spatial memories can be classified due to their persistence after initial learning and recall. Memories that only persist for short periods of time are called short-term memories (STM). In human studies, this classification is attributed to temporary formation of memories required for task performance in the range of seconds to minutes, while working memory refers to the manipulation of short-term memories^54^. In rodent studies, this classification is particularly different, as working memory refers to temporary memory formation required for task performance in the scale of seconds to minutes, and STM refer to storage of information that endure for minutes to several hours. This distinction is relevant when comparing clinical studies with research using animal models. Specific types of STM seem to be somewhat resistant to decay during aging. Particularly, when learning and recall sessions are paired temporarily close together, older individuals and rodents are able to recall tasks, but show increasing errors as the delay increases^55–57^. This delay-specific impairment in STM is mostly not accounted in the design of human and rodent studies, which might result in incomplete conclusions when memory performance is evaluated to only account for short or long intervals.

Activity between several brain regions during long periods of time is required for memory consolidation and consequent formation of long-term memories (LTM). The formation of LTM is prone to disruption during aging in humans and rodents^58–62^. Recollection of LTM formed years or decades ago in humans is largely spared, which suggests that aging mostly impacts the acquisition of new information, which would be consolidated into LTM, but not the retrieval of LTM. One recent study evaluated healthy aged individuals´ performance in a cognitive examination over a period of 30 min or 4 weeks. They found that while at the 30 min test individuals showed overall good performance, 4 weeks later they detected accelerated long-term forgetting^63^. These findings indicate that evaluating long-term cognitive performance is a sensitive way of assessing age-related cognitive decline. Clinical and animal studies usually do not evaluate such long-term memory deficits. These studies mostly focus on age-dependent changes associated with the formation of recent memories. Moreover, longitudinal studies that accompany memory decay from middle to advanced age are underperformed in rodent studies. This is in part due to the requirement of inducing learning in middle-aged rodents and assessing memory recall after their age advances, which are time-consuming experiences to perform. As discussed in the next sections, most of the behavior paradigms used do not induce memories that would persist for several months. One exception would be tests that form associative memories based on aversive stimuli. It is not advised to retrain animals several times in cognitive tasks, as this process might introduce confounds of memory reconsolidation processes and/or reinforcement due to the repeated exposure to learning cues. A detailed discussion of behavior paradigms that can be used for these studies is presented in section 2.3 Cognitive tests used to evaluate age-related cognitive decline and is summarized in Fig. 2 and Table 1.

**Table 1:** Summary of most common cognitive tests used in clinical and animal studies.

Cognitive test	Main application	Type of cognitive evaluation	Main brain regions evaluated	Main advantages	Main disadvantages
Mini-Mental State Examination	Humans	General cognitive performance	Limbic system	Easy to use in clinical practice; sensitive for dementia	Education level and gender influence score; low sensitivity for mild forms of cognitive impairment
Montreal Cognitive Assessment	Humans	General cognitive performance	Hippocampus and cortical regions	Easy to use in clinical practice; sensitive for mild forms of cognitive impairment	Intellectually more challenging
Fear conditioning	Rodent models	Associative memory	Amygdala Hippocampus Auditory cortex	Reliable protocols that allow memories to be evaluated for long periods; single learning session	Aversive stimulus can introduce bias in aged studies; difficult to implement in human studies
Delay eyeblink conditioning	Rodent models and humans	Associative memory	Cerebellum	Impairments associated to ageing are detected in humans and rodents	Possible cofounds of age-dependent hearing loss
Morris water maze	Rodent models and humans	Spatial learning	Hippocampus, striatum, cerebral cortex and cerebellum	Allows the study of allocentric and egocentric navigation; virtual reality analogues available for humans	Time consuming protocols; required multiple training sessions; induces some degree of stress.
Barnes maze	Rodent models	Spatial learning	Hippocampus Prefrontal cortex	Sensitive for early age-associated cognitive impairment; induces less stress than the Morris water maze	Not as widespread as the Morris water maze; no current available analogue test in humans
Object location test	Rodent models and humans	Spatial learning	Hippocampus Prefrontal cortex	Controls for reduced motor activity; analogue tests available for humans	The dependence on particular brain regions varies according to the protocol used

### Aging-related functional changes in the brain

There is extensive evidence of structural changes during aging in the brain, such as loss of synaptic complexity and reduced white matter volumes^21,31^. These alterations are accompanied by functional impairments that hinder brain plasticity and function.

In this section, we focus on known functional alterations that occur in the aging brain. Memory formation and recall recruit activity of several brain regions, which are altered during aging. Early studies suggested that aged individuals show a decrease in neuronal activation and recruit different brain regions compared to young individuals^64–66^. Nonetheless, these initial findings did not consider that young individuals tend to outperform aged individuals, which might lead to different patterns of brain activity independently of aging. Further work addressed this issue by separating aged individuals based on their performance in a spatial memory task and comparing their patterns of brain activity to young individuals^67^. The authors found that aged good performers and young individuals displayed similar patterns of brain activity evaluated by blood oxygen level-dependent signal. These findings indicate that patterns of activity predict the performance level in cognitive tasks and that activity may become less specific with age (Fig. 2).

One complementary hypothesis to altered activity patterns is the posterior-to-anterior shift in aging (Fig. 2). This theory postulates that during aging there is recruitment of anterior brain regions, such as the prefrontal cortex, as a compensation for impairments in posterior regions^68^. A recent study applied a novel model-based multivariate analysis to understand whether overactivation of the prefrontal cortex in aged individuals was a compensation mechanism or a recruitment impairment^69^. The authors found that increased prefrontal activity was associated with less specific or less efficient cognitive outcomes. Similar findings have been observed in aged rodents, particularly cognitively impaired aged rats show abnormal activation of cortical regions and subregions of the hippocampus^70,71^. Similarly, to humans, this pattern of overactivation was not observed in aged cognitively unimpaired rats, indicating an unspecific recruitment of other brain regions.

Besides the hippocampus and cortex, motor-associated regions are also affected with aging (Fig. 2). One study indicated that after learning a motor task, older adults showed an increased activation of several cortical regions, but also the cerebellum compared to young participants^72^. A more recent study that evaluated brain activity during spatial navigation, also observed increased cerebellar activity in aged adults^73^. Conversely, another study found that cerebellar activity is compromised in aged adults during motor learning^74^. These apparent contradictory results point to altered cerebellar function, which is in agreement with other studies^75–77^. Overall, these findings indicate that brain activity is altered during aging in several regions such as the cortex, hippocampus and cerebellum although more studies are required to evaluate task-specific alterations.

### Cognitive tests used to evaluate age-related cognitive decline

#### Questionnaire-based tests

There is an extensive collection of cognitive tests used in human and rodent studies. Here we explore some of the most commonly used tests by clinicians and behavior neuroscientists. One of the most common tests used worldwide, mainly by clinicians, is the Mini-Mental State Examination (MMSE)^78^ (Fig. 2 and Table 1). This questionnaire-based assessment consists on a set of questions that are used to screen for dementia and cognitive decline in the elderly. One of the main advantages of this test is that it provides a fast assessment of cognitive function without extensive training. Moreover, it has been reported to be sensitive for dementia, as diagnosed patients with dementia typically show an annual 3-point decline in MMSE score^79^. In clinical practice, the MMSE is used to evaluate cognitive performance as a whole, although performance in this test has been shown to correlate with atrophy of particular brain regions in the limbic system^80^. However, there are several factors that might influence the absolute score of this test, such as education level and gender^81^. Another limitation of this cognitive assessment is that it lacks sensitivity for milder forms of cognitive impairment^78,82^. For these reasons attempts have been made to develop alternatives to the MMSE. Developed in 2005, the Montreal Cognitive Assessment (MoCA) is one of such alternatives and since then has become widespread in clinical practice^83^ (Fig. 2 and Table 1). The MoCA has proven to be sensitive enough to detect mild forms of cognitive impairment, while simultaneously detecting dementias associated with neurodegenerative disorders such as Alzheimer’s Disease^84^. Moreover, recent evidence suggests that performance scores in this test correlate with structural alterations of the hippocampus and cortical regions^85^. Components of the MoCA test are based on recollection of words over periods of time, which make it sensitive for detecting memory and attention deficits. It does present some limitations, as the test partially relies on fine motor movement, which might introduce bias into the score. Moreover, compared to the MMSE, the MoCA is an overall more intellectually demanding set of tests, and adjustments for factors such as education are needed^86^. Besides their sensitivity for dementia studies that evaluated the cognitive status of healthy aged adults using the MMSE and MoCA has shown that these tests might be useful for age-related cognitive decline spared of disease^87,88^. Although not without limitations, both tests can be powerful tools for an initial clinical evaluation of cognitive performance of the elderly. Particularly, they do not require long periods of time for preparation and can therefore be used to track the performance of patients during routine consultations over several years. As discussed above, cognitive aging affects specific types of memory, therefore although the MMSE and MoCA have clinical relevance, their broad type of evaluation does not provide much insight on specific types of age-related cognitive decline. Other alternatives have also been shown to be sensitive to detect age-related cognitive deficits such as automated computer tests^89^. Altogether these tests can be used in combination to allow more detailed evaluation particularly with virtual reality based on rodent studies^90,91^.

#### Behavior paradigms to evaluate age-related cognitive decline

In the past decades, clinical researchers have used an array of tasks that can assess deficits in spatial memory formation and navigation strategies using rodent models^92–94^. These paradigms have been used to evaluate cognitive function in physiological conditions and neurological disorders. Moreover, several tests have been established or adapted to study age-related cognitive decline. Criteria have been proposed for assessing the sensitivity of behavior tasks to study cognitive decline during aging^95–97^. Particularly, behavior rodent tasks should: (1) be sensitive to detect memory deficits, when comparing old and young animals; (2) avoid bias from other processes that might affect the outcome of the test; (3) avoid the use of dietary restrictions or stressors that might hinder the performance of aged animals; (4) detect memory deficits in young animals, when lesions occur brain regions that model deficits observed in aged animals; (5) be consistent across several animal strains and multiple species, such as humans and rodents; (6) be sensitive to established pharmacological approaches that are known to improve behavior in clinical trials with aged humans and; (7) depend on brain regions that support learning and memory formation. Based on these criteria, over the years several behavior paradigms have been developed that can, to some extent, be applied to rodents and humans. We will expand on tests that rely on spatial associative learning based on aversive or non-aversive cues.

##### Behavior paradigms dependent on conditioning

Associative learning relies on pairing between two independent stimuli as a measure of learning and memory. In classical conditioning paradigms, a neutral stimulus such as a chamber (contextual fear conditioning) or sound (cue fear conditioning) is presented in combination with an aversive stimulus (foot-shock) to induce associative learning (Fig. 2 and Table 1). After this initial learning session, the neutral stimulus is presented in the recall sessions in isolation. The readout of learning in these tasks relies on freezing behavior (absence of movement apart from breathing). One of the advantages of these tests, is that the learning event is clearly defined in time, during the association session, and can be separated from the recall session, without confounds of multiple training sessions. Another advantage is that the intensity and/or number of shocks can be modulated to induce memories that can potentially last several months. This might be particularly useful to understand how associative memories formed in middle age might be affected during aging. As the basis of these tests relies on aversive forms of learning generally rodents show consistent ability to freeze, though this might introduce bias when comparing young and older animals. Indeed, it is often reported that aged mice and rats do not show general impairments in freezing behavior, which can possibly be attributed to altered anxiety responses to the aversive stimuli^98,99^. Variations of these tasks, that heavily rely on intact hippocampal function, such as trace fear conditioning are more sensitive in detecting spatial memory deficits in aged rodents^100^. In humans, it is challenging to establish fear conditioning protocols that model studies in rodents^92^.

Another associative task used to evaluate age-related memory deficits is delay eyeblink conditioning (Fig. 2. And Table 1). In this task, animals are trained to associate a neutral stimulus (e.g., tone) with a blink reflex-emitting stimulus (i.e., air puff to the eye) to elicit an unconditioned response (i.e., eyeblink). In contrast to other behavior tasks discussed in this review, delay eyeblink conditioning is dependent on the cerebellum, which is affected by the aging process. In accordance, numerous studies have demonstrated that aged adults show fewer conditioned responses than young adults^101–103^. Aged rodents also show impairments in this task, similarly to humans. One study compared young and aged mice that have intact auditory function in contextual or trace fear conditioning and eyeblink classical conditioning. They found that aged mice had impairments in eyeblink associative learning, although no age-related differences were observed in fear conditioning paradigms^75^. The authors concluded that cerebellar functions show earlier signs of senescence compared to the hippocampus. One possible limitation of this paradigm is that depends on an auditory tone as a neutral stimulus. It has been proposed that there is an age-dependent decrease in auditory function, which can potentially introduce bias^104^.

As discussed, fear conditioning paradigms might introduce confounds, due to their dependence on brain regions besides the hippocampus and altered anxiety-like behavior. An alternative to these studies is trace fear conditioning, which is accepted as a more sensitive test to evaluate hippocampal function in aged mice^100,105^. This variation of classical fear conditioning introduces a trace period that separates each conditioning trial, which increases the role of connections between the medial prefrontal cortex and the hippocampus for memory^106^. Nonetheless, these findings are consistent with the sensibility of eyeblink classical conditioning to evaluate cognitive decline associated with cerebellar dysfunction, which is consistent with other animal studies^97,107^. The combination of behavior paradigms evaluating different brain regions that are required for associative learning is useful to track age-related differences over time. The paradigms discussed so far induce association between fear responses and spatial component or reflexes. Therefore, it is possible that they can introduce unwanted confounds. These associative tasks highly depend on hippocampal (contextual/trace fear conditioning) or cerebellar function (delay eyeblink conditioning), regions that are known to be mostly affected during aging. Therefore, the use of these tasks, particularly in rodents are useful to investigate cellular and molecular alterations associated with age-related dysfunction in these regions.

In the next section, we will layout alternative strategies that are not based on Pavlovian conditioning.

##### Behavior paradigms based on spatial navigation

In 1981, Richard Morris developed a spatial behavior task based on a water-maze for rats^108,109^. Since then, the Morris water maze has become one of the golden standards for evaluating spatial learning and memory performance in rodents (Fig. 2 and Table 1). This paradigm consists in training animals to learn the spatial localization of a hidden platform in a water-maze over several trials. Animals learn this location using reference cues that are displayed around the maze, therefore eliciting spatial memory formation. Initially developed for rats, this task has been validated for mice, although their performance is generally poorer^110^. Aged rodents trained in the Morris water maze consistently display deficits in memory formation, compared to young^111–114^. One of the advantages of this test is the ability to evaluate allocentric and egocentric navigational strategies. Young animals that learn the spatial location of the hidden platform tend to use allocentric navigation, based on the spatial cues presented during the learning trials, while aged animals adopt egocentric navigation patterns as an alternative strategy to find the platform^53^. These findings are in accordance with aging human studies that evaluate spatial memory performance. The wide-spread use of the Morris water maze has encouraged the development of virtual versions that can be applied to humans^115^. Aged individuals spend more time using proximal navigation cues (egocentric) in comparison with young participants that navigate by reference to cues present in the room (allocentric). These findings have been replicated in other studies demonstrating that older adults display a robust shift for egocentric performance^116^. Similarly, another study using a real-space analogue of the Morris water maze found selective impairments in allocentric navigation in aged individuals, but preserved egocentric strategies^117^. In this study, however, they did not detect any differences in overall memory performance between the different age groups, which might suggest that a shift between allocentric to egocentric strategies might compensate performance in these tasks^117^. Recent findings separated aged good from poor performers and evaluated navigation strategies using virtual versions of the maze. The authors speculated that aged poor performers might be unable to adapt new navigation strategies to compensate altered neuronal subtracts^118^. Future human and rodent studies should couple navigational strategy and performance with imaging, to understand the engagement of distinct brain regions in allocentric and egocentric strategies during aging. For detailed reading on age-related egocentric and allocentric alterations during aging we recommend this systematic review^49^. This evaluation would represent a significant advancement in the field to develop target therapies for brain region specific impairments during aging. The Morris water maze depends on swimming-induced stress as a motivator for escaping, therefore age-associated changes in stress response might bias performance^119^. Moreover, cerebellar age-associated dysfunction can hinder swimming performance of aged rodents and influence performance, independently of altered memory capacity.

An alternative to the Morris water maze was developed by Carol Barnes in 1979. This paradigm consists in rodents learning the location of a target hole for escape based on distal cues. This task is dependent on prefrontal and hippocampal function^120^. Contrarily to the Morris water maze this task evaluates spatial memory formation without major aversive stimuli as it is a dry maze. Nonetheless, both tests induce an increase in the levels of stress hormones compared to naïve animals, but corticosterone levels are significantly higher in the Morris water maze compared to the Barnes maze^121^. Therefore, the Barnes Maze is accepted to be sensitive for spatial memory deficits, but requires less physical effort. This test was initially developed to evaluate age-related cognitive performance in aged rats that show impaired performance^121^. Versions of this task have also been applied to study aged mice^28^. Over the years it has shown to be a reliable tool to access memory performance during aging (Fig. 2 and Table 1). A recent study evaluated performance in the Barnes maze from young adulthood to middle age^122^. The authors found that mice aged 8–12 months old already show spatial memory impairments supporting the sensitivity of this behavior paradigm. In humans; however, there are currently no analogues to the Barnes maze. Nevertheless, the sensitivity of this test in rodent models should encourage future clinical research to develop similar paradigms that can be applied to humans. During the writing of this review, another behavior task was developed to evaluate spatial navigation in rats and humans^123^. This open-field navigation task, called Tartarus maze, allows spatial navigation pattern analysis. Moreover, the authors set up a physical apparatus that is applied to rats and an analogue system based on immersive head-mounted display virtual environment applied to humans. Importantly, navigation strategies used by both species showed strong similarities, which allows future direct comparisons that can potentially be used in aging studies in the future.

The last behavior protocol widely used in rodent animal models discussed in this review is the object location test. This paradigm also evaluates spatial learning and memory and combines several advantages to the tests described so far. In this test, animals explore an arena that contains novel objects. Due to their natural novelty seeking behavior, rodents will explore the objects during the learning phase and form a spatial map of their localization in the arena. Later, one of the objects is displaced onto a new location in the recall session, and animals that successfully learn this task will spend more time exploring the displaced compared to the non-displaced object. Aged mice show memory impairments in this task as they explore equally the displaced and non-displaced objects^124^ (Fig. 2 and Table 1). One of the main advantages of using this paradigm is that it controls for reduced motor activity and alteration in stress responses that accompany aging^125^. This is achieved since the time spent exploring the displaced object can be normalized for the total time of exploration of each animal. In the clinical setting it is well established that object-associated memories are affected in the elderly^126,127^. Particularly, a study evaluated the memory performance of aged and young adults for sequences of object–location associations in combination with brain imaging^128^. They found that decreased performance by older adults was associated with fronto-striatal network and left superior temporal lobe activity, compared to young participants that use posterior brain regions. These findings support the idea that the elderly recruit alternative neuronal networks to perform in spatial-associated tasks. Nonetheless, these deficits seem to be selective, as aged adults show difficulties in distinguishing the location of objects in space, but retain the ability to recognize the objects presented^129^. Altogether many of these tasks that were initially designed and developed for rodent models, have analogues for use in the human clinical setting, the main exception being the Barnes Maze. The consistency of findings reflects the conserved nature of spatial memory impairments observed during cognitive aging across species.

#### Virtual-reality-based assessment of cognitive abilities

Novel technological advances allow the development of user-friendly tasks that can be easily implemented into clinical setting. Particularly, the emergence of virtual-reality-based tests that analyze spatial performance are novel powerful tools that can potentially bridge findings from human studies and animal models^130^. Indeed, a recent study suggested that aged individuals show impairments in navigation in virtual mazes^131^. Particularly, older and young adults experienced nine learning trials in a virtual maze, based on several spatial cues. Once these cues were removed, older adults used mainly egocentric navigational strategies, compared to young adults that used mostly allocentric negational strategies. Similar findings have been observed using other virtual-reality mazes^118^. The use of a virtual mazes brings the advantage of eliminating non-specific cues and the human factor present in the environment where non-virtual tests are performed. Moreover, besides the possible diagnosing capacity of these tests, evidence indicates that the use of virtual-reality-based tasks can improve cognitive abilities of aged individuals over time^132^. These virtual-reality tools have only recently been applied to laboratory rodent models. Although studies that use virtual reality tools to understand age-related cognitive decline in rodents are sparse, some efforts have been made^133^. For example, researchers used a virtual maze to train adult rats to understand the mechanisms associated with spatial navigation and memory formation. Using this paradigm, the authors demonstrated a direct involvement of activation of the N-methyl-D-aspartate receptor (NMDAR) for navigation in this virtual maze, similarly to what has been shown in mice and human studies^134,135^. In summary, there is a wide-range of behavior tests available in clinical settings and in behavior laboratories. These evaluate different components of age-related cognitive decline and therefore can be used in combination to understand alterations associated with specific brain regions. The development of virtual-reality-based tests shows a high potential for future standardization of screening aged adults in the clinics without the need for spatial tasks that are difficult to implement in a clinical setting. Moreover, these methods can potentially generate comparable tasks to be developed for animal models which might bring closer together the data generated in preclinical and clinical studies.

### Cognitive tests used as inclusion criteria for clinical trials in age-related cognitive decline

**Fig. 3:**
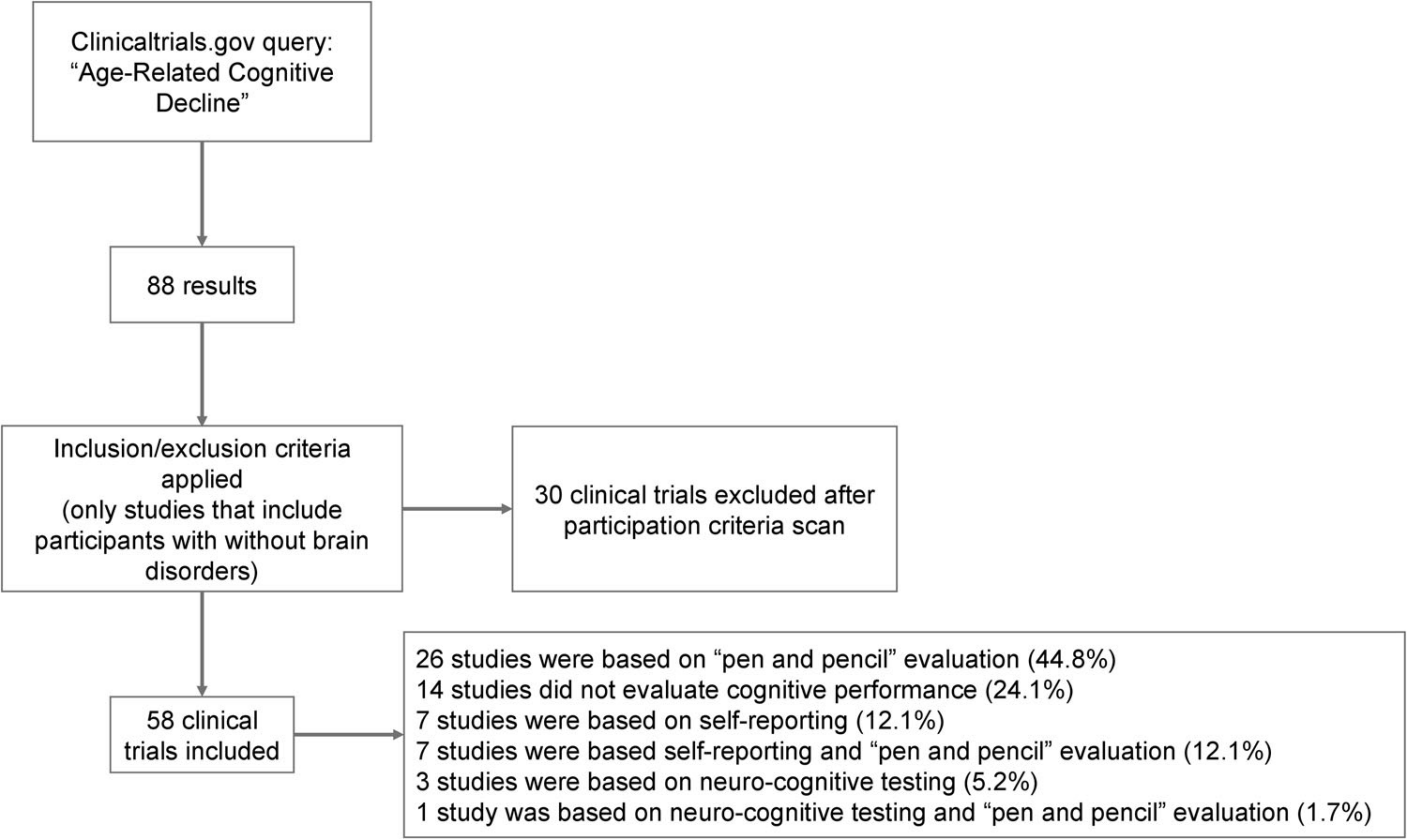
Flow chart of the systematic approach. The diagram depicts the flow of information through the different phases of screening.

In the previous sections, we discussed common tests to evaluate cognitive performance during aging in mice and humans. Several tests show selective sensitivity, which is useful to discriminate specific impairments associated with aging. Therefore, it would be reasonable that clinical trials used as inclusion criteria the “best” possible paradigms to evaluate novel approaches to delay/treat deterioration of cognitive abilities during aging. We characterized the available clinical trials available in the clinicaltrials.gov under the category of “Age-Related Cognitive Decline” (Fig. 3). Only trials that included memory deficits without dementia were considered, a total of 58 clinical trials were considered eligible out of the initial 88 results screened. Most of these studies show common inclusion categories for selection of cognitively impaired participants. The majority of trials used screening based on “pen and paper” cognitive testing such as the MMSE or MoCA tests (44.8%). As previously discussed, these tests are widespread in clinical practice. Nonetheless, they lack the specificity to evaluate particular components of cognition in affected in patients. This might lead to the recruitment of aged individuals that have low scoring in these tests, but have age-related cognitive decline associated to different brain regions/tasks. In this scenario, even a therapeutic approach that might prove effective for one component could produce negative effects if there is recruitment bias. A second strategy is the use of self-identified age-related cognitive decline (12.1%). This criterion, which consists on self-reported worsening or increase of memory loss, might not be accurate enough to differentiate among the heterogeneity of individuals that display cognitive deficits. A third series of trials use neuro-cognitive testing to screen for cognitive impairments (5.2%). Moreover, a minor proportion of studies involved a combination of these strategies (13.8%) and a significant proportion did not perform cognitive evaluation prior to recruitment (24.1%) (Fig. 3). Overall, the wide toolkit of sensitive age-related cognitive tests available such as virtual environments or analogue tests to animal paradigms are largely not applied for screening the elderly in clinical trials.

## Ending remarks and open questions

In this review, we discussed recent findings on altered brain activity during aging in humans and rodent models. We pointed to the main strategies to diagnose/investigate age-related cognitive decline and we contrasted their use in clinical settings and the laboratory. Lastly, we interrogated whether there was a wide variety of tests being currently used to select elderly patients for clinical trials enrollment. Many behavior paradigms have been successfully developed and most are used in rodent’s animal models and humans. The interdisciplinary versatility of these tests is crucial to develop therapeutic approaches that can be applied to patients that suffer from age-related cognitive decline. Moreover, these tests can also be used to understand the circuit and molecular alterations in cognitive abilities that affect the elderly. The spread of virtual-reality based tests is an opportunity to implement reliable and sensitive testing in the clinics and laboratory. Finally, clinical scientists should take advantage of the availability of sensitive cognitive tests beyond the classic “pen and paper” strategies particularly for admission into clinical trials.
